# Idiopathic Hypereosinophilic Syndrome With Cutaneous Manifestations: A Case Report

**DOI:** 10.7759/cureus.76638

**Published:** 2024-12-30

**Authors:** Nicole Foreman, Ana Neves, João Rocha, Manuela Dias, Jorge S Almeida

**Affiliations:** 1 Intensive Care, Unidade Local De Saúde De São João, Porto, PRT; 2 Internal Medicine, Unidade Local De Saúde De São João, Porto, PRT

**Keywords:** corticotherapy, cutaneous manifestations, hypereosinophilia, hypereosinophilic syndrome, rheumatoid arthritis

## Abstract

Hypereosinophilic syndrome (HES) is marked by eosinophilic infiltration and the release of inflammatory mediators that cause damage to multiple organs. Despite careful evaluation of hypereosinophilia, the etiology of most cases remains undefined. Eosinophils may cause damage in almost all organs, and most patients present with dermatological manifestations. We report a case of a 70-year-old man with a history of rheumatoid arthritis, who is under treatment with rituximab and leflunomide. He presented with generalized erythematous and pruritic cutaneous lesions that became scaly and hypopigmented, some of which were ulcerative and infected. Peripheral blood hypereosinophilia was noted (>6.0x10^9^ eosinophils/L). Primary and secondary causes of hypereosinophilia, including neoplasms and infections, were excluded, and idiopathic HES was assumed. The patient was started on oral prednisolone with complete resolution of the lesions.

## Introduction

Hypereosinophilic syndrome (HES) is a rare condition characterized by hypereosinophilia (>1.5×10^9^ eosinophils/L in peripheral blood) with consequent organ damage [1,2]. HES can be classified into three different groups: primary/neoplastic (due to stem cell, myeloid, or eosinophilic neoplasms), secondary/reactive (due to excessive eosinophilic stimulation by cytokines produced in response to parasitic infections, solid tumors, or T-cell lymphomas), and idiopathic [2].

Organ damage patterns mediated by eosinophils may be heterogeneous, affecting mostly the skin, but also the lungs, heart, gastrointestinal, and nervous system [3]. There is a broad spectrum of cutaneous presentations, such as eczema, erythroderma, lichenification, angioedema, and urticaria [4,5]. Data on HES in patients undergoing maintenance B-cell-depleting treatment, such as rituximab, are scarce. In this article, we present an atypical cutaneous presentation of HES in a patient under chronic treatment with rituximab.

## Case presentation

**Figure 1:**
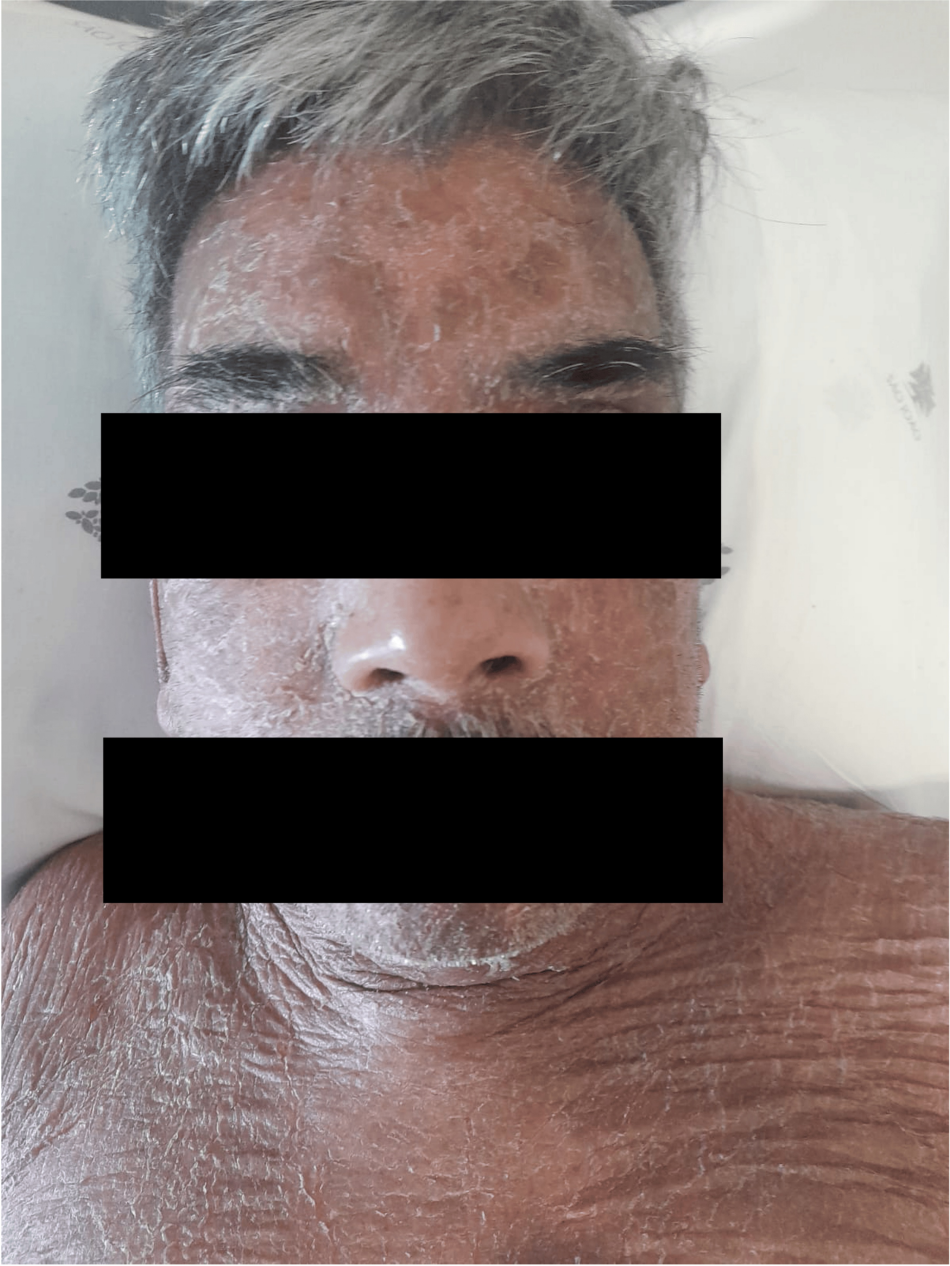
Scaly and erythematous cutaneous lesions on the face and upper thorax

**Figure 2:**
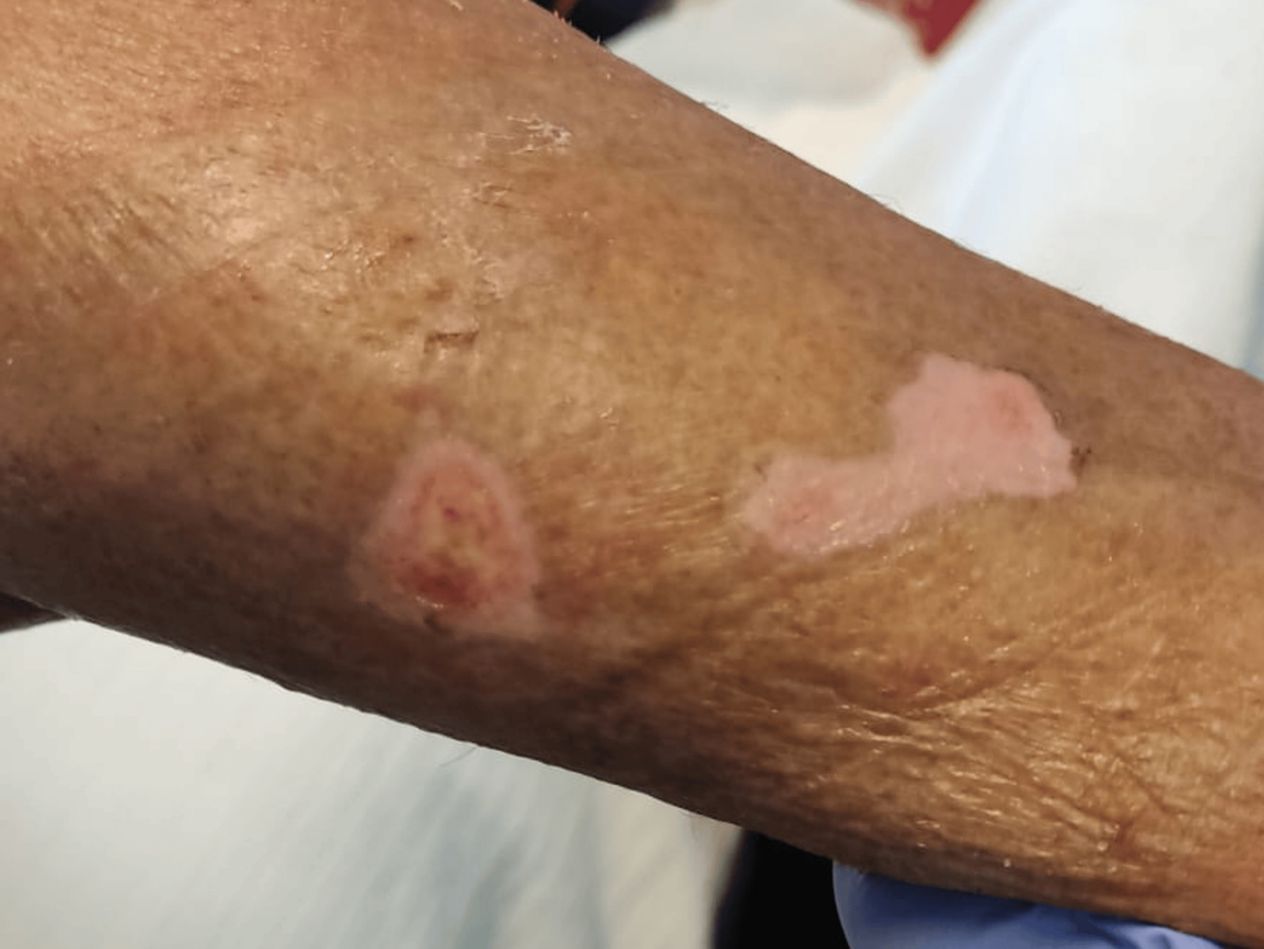
Hypopigmented lesions that later became ulcerative

**Figure 3:**
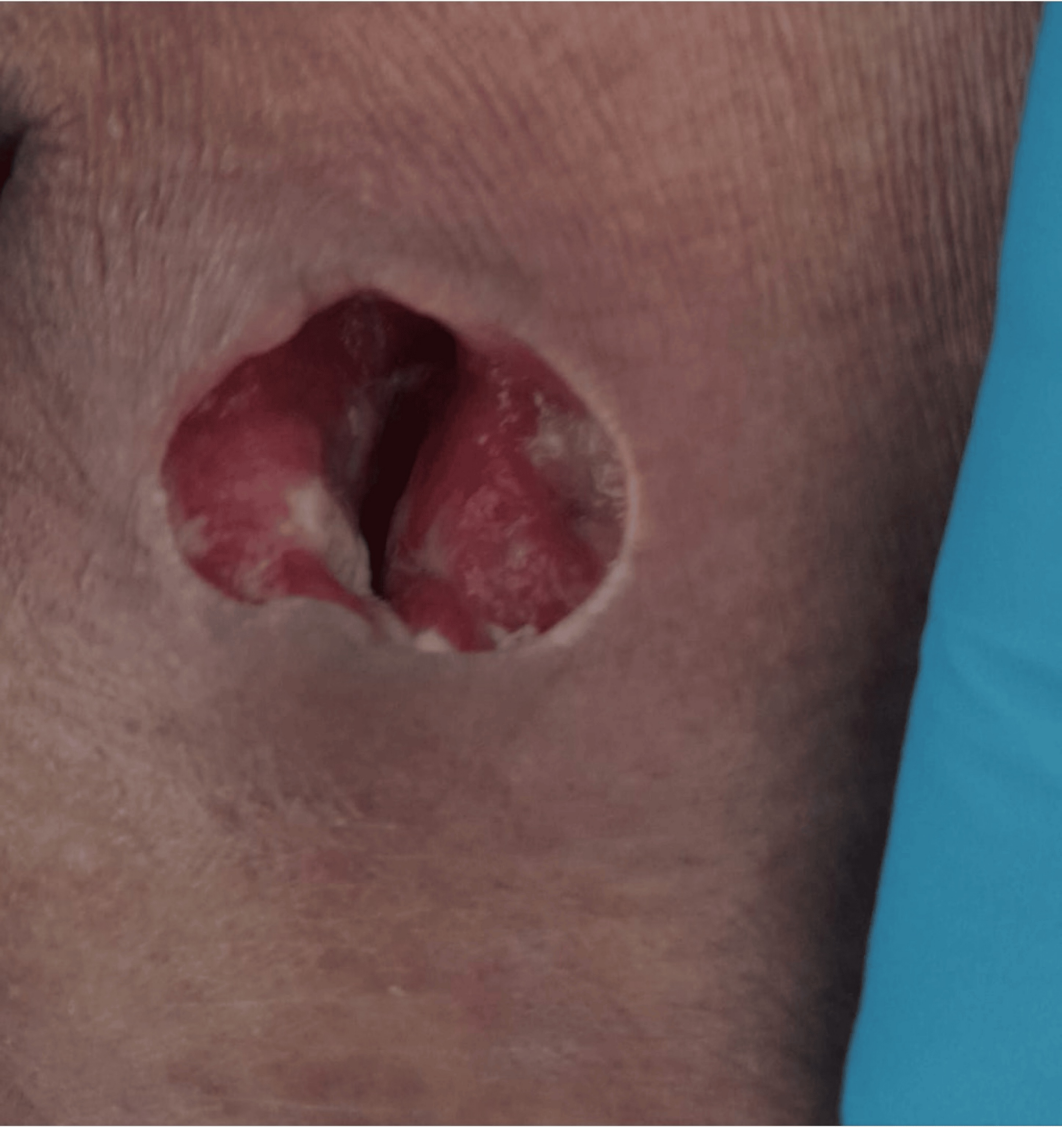
An ulcerative lesion with purulent drainage in the left scapula

A 72-year-old man from India, staying in Portugal for vacation, presented in the emergency department of our hospital with a four-week history of cutaneous lesions. Initially, these lesions were pruriginous and erythematous (Figure 1), turning scaly and hypopigmented (Figure 2), with distribution throughout the body, including the palms, soles, and face, but sparing oral and genital mucosa. Some lesions became ulcerative in several regions, particularly in the left scapula, with exposed muscle and purulent discharge (Figure 3). Three weeks before admission, he was given cetirizine 20 mg/day and deflazacort 30 mg/day, tapered over one week. Although initial improvement was observed, some of the ulcerated lesions started to present purulent discharge, so a course of amoxicillin-clavulanate was given. An improvement of the purulent drainage was noted; however, the erythematous lesions kept progressing into the upper and lower limbs, trunk, and face. No other complaints were reported, particularly constitutional symptoms. At examination, fever and bilateral conjunctival erythema were also noted. No other relevant alterations were found in the physical examination. 

The patient had rheumatoid arthritis, currently treated with deflazacort 3 mg/day, leflunomide 20 mg/day, and rituximab 500mg every six months, with clinical remission. Rituximab was initiated in November 2022, and the last administration was given three weeks before the presentation. His chronic medication also included calcium and vitamin D supplementation. No contact with animals or apparently sick people was reported, and he did not travel to other countries besides Portugal. He denied any ingestion of non-pasteurized dairy products, non-potable water, or non-cocked food. His vaccination status was unknown. Six months earlier, he performed an upper endoscopy and colonoscopy, with no alterations described.

The patient was admitted to the Internal Medicine ward for treatment and further study. Table 1 summarizes the analytical findings documented during the diagnosis workup. Laboratory tests were remarkable for leukocytosis with significant hypereosinophilia (6.13x10^^9^/L), elevated acute phase reactants, slight normocytic and normochromic anemia, hypogammaglobulinemia with increased immunoglobulin E (229 mg/dL), and elevated lactate dehydrogenase. 

**Table 1:** Laboratory tests at admission and discharge CRP: C-reactive protein; ALT: alanine transaminase; AST: aspartate transferase; GGT: gamma-glutamyl transferase; CK: creatine kinase; BNP: B-type natriuretic peptide; RF: rheumatoid factor; CCP: cyclic citrullinated peptide; ANA: antinuclear antibody; dsDNA: double-stranded DNA; ANCA-PR3: anti-proteinase 3 antineutrophil cytoplasmic antibody; ANCA-MPO: Myeloperoxidase-antineutrophil cytoplasm antibody; EVA: extractable nuclear antigen; HBsAg: Hepatitis B surface antigen

	Reference values	Admission (day one)	Discharge (day 32)
Hemoglobin (g/dL)	13.0-18.0	11.6	10.9
Mean corpuscular volume (fL)	87.0-103.0	87.6	89.5
Mean corpuscular hemoglobin concentration (g/dL)	28.0-36.0	31.1	30.4
Leucocytes (x10^9^/L)	4.0-11.0	25.74	6.02
Neutrophils (%)	53.8-69.8	16.96	72.5
Lymphocytes (%)	22.6-36.6	1.80	17.9
Monocytes (%)	4.7-8.7	0.51	8.1
Basophils (%)	0.0-1.5		0.5
Eosinophils (%)	0.6-4.6	6.13	0.7
Platelets (x10^9^/L)	150-400	465	193
Erythrocyte sedimentation rate (mm/1^st ^hour)	0-20	21	-
Iron (µg/dL)	53-167	95	-
Transferrin (mg/dL)	200-360	132	-
Transferrin saturation (%)	20-50	51	-
Ferritin (ng/mL)	-	1252.2	-
Haptoglobin (mg/dL)	50-320	185	-
Direct Coombs Test	-	Negative	-
Peripheral blood smear	-	Normal	-
Urea (mg/dL)	10-50	47	81
Creatinine (mg/dL)	0.67-1.17	1.02	0.94
Sodium (mEq/L)	135-147	153	142
Potassium (mEq/L)	3.5-5.1	4.4	3.9
Chlorides [(mEq/L)	101-109	121	105
Calcium (mEq/L)	4.1-5.2	4.3	-
Magnesium (mEq/L)	1.55-2.05	1.67	-
Lactate dehydrogenase (U/L)	135-225	525	249
CRP (mg/L)	<3.0	54.8	5.4
Total proteins (g/L)	64.0-83.0	53.5	-
Albumin (g/L)	38.0-51.0	29.4	-
ALT (U/L)	10-37	39	26
AST (U/L)	10-37	28	13
Alkaline phosphatase (U/L)	30-120	129	117
GGT (U/L)	10-49	37	105
Total Bilirubin (mg/dL)	<1.20	0.47	0.45
Myoglobin (ng/mL)	<146.9	-	-
CK ( U/L)	10-172	108	
Troponin (ng/L)	<34.0	12.9	-
BNP (pg/mL)	<100.0	109.3	-
Immunoglobulin G (mg/dL)	600-1560	464	-
Immunoglobulin A (mg/dL)	90-410	64	-
Immunoglobulin M (mg/dL)	30-360	27	-
Immunoglobulin E (kU/L)	<114	229	-
RF (UI/mL)	<30.0	<9.7	
Anti-CCP (U/mL)	<7.0	<0.4	-
ANA	-	Negative	-
Anti-dsDNA (UI/mL)	<100.0	<10.0	-
ANCA-PR3 (U/mL)	<20	<2	-
ANCA-MPO (U/mL)	<20	<2	-
ENA screening	-	Negative	-
Angiotensin-converting analysis (U/L)	20-70	46	-
Tryptase (µg/L)	<11.40	8.17	-
Serum amyloid A protein (mg/L)	0.0-6.4	7.7	-
Serum protein electrophoresis	-	No monoclonal spike	-
Human immunodeficiency virus 1+2 (antigen+antibody*)*	-	Negative	-
HBsAg	-	Negative	-
Hepatitis C antibody		Negative	-
Syphilis test		Negative	-
Bacteriological, myco-bacteriological, and mycological examination (blood)	-	Negative	-
Bacteriological and parasitological examination (urine)	-	Negative	-
Parasitological examination (stool)	-	Negative	-
DNA *Giardia lamblia, Entamoeba histolytica, Cryptosporidium, Blastocystis hominis, Dientamoeba fragilis, Cyclospora cayetanensis* (stool)	-	Negative	-
Cytomegalovirus	-	Immune	-
Parvovirus B19	-	Immune	-
Toxoplasmosis	-	Nonimmune	-
Varicella	-	Immune	-
Mycoplasma pneumoniae	-	Nonimmune	-
Epstein-barr virus	-	Immune	-
Borrelia	-	Nonimmune	-
Chlamydia pneumoniae	-	Nonimmune	-
Coxiella burnetii	-	Nonimmune	-
Rickettsia conorii	-	Nonimmune	-
Wright reaction	-	Negative	-
DNA *Aspergillus fumigatus*	-	Negative	-
DNA* Burkholderia pseudomallei*	-	Negative	-
DNA Strogyloides	-	Negative	-
DNA Toxocara	-	Negative	-

The subsequential investigation was directed to the differential diagnosis of hypereosinophilia and cutaneous lesions. A peripheral blood smear was made, which showed no pathological findings. Serum tryptase and cyanocobalamin were normal. Peripheral blood flow cytometry of T lymphocytes revealed no evidence of monoclonality, and immunophenotyping showed a low B cell count and no changes in the T cell lineage. A bone marrow biopsy and aspirate were also made, with no evidence of malignancy in pathological examination, and immunophenotyping only revealed decreased B cell counts. However, a small eosinophil infiltrate was noted. Screening of the FIP1L1-PDGFRA mutation was negative. Extensive microbiological studies from peripheral blood, bone marrow, urine, feces, and cutaneous exudate were negative, including cultures for mycobacteria and parasites. Immunological studies were negative, particularly antineutrophil cytoplasmic antibodies. Two skin biopsies of the described lesions were performed, both showing hyperkeratosis and inflammatory infiltration with some eosinophils and without vasculitic lesions.

An evaluation of occult neoplasms and potential organ damage induced by eosinophilic infiltration was also made. Chest radiography showed no abnormal findings. Full body computed tomography was performed, with no apparent neoplastic lesions; however, some nodules/micro-nodules of ground glass in the lungs were noted. A bronco-alveolar lavage was made, which was positive for *Haemophilus influenzae*. The remaining microbiological results, including for *Mycobacterium tuberculosis*, and cytology for malignant cells were both negative. The electrocardiogram showed no cardiac rhythm alterations, and transthoracic echocardiography showed no cardiac functional or structural abnormalities. Ophthalmology examination excluded ocular involvement, without signs of vasculitis or thromboembolic events.

At admission, piperacillin/tazobactam plus vancomycin were empirically started, with later adjustment to ceftazidime to cover a possible *Burkholderia pseudomallei* infection. Two doses of ivermectin were also administered. However, neither strategy led to significant clinical improvement nor an analytical response in terms of total eosinophil count and acute phase reactants. Suspecting HES, prednisolone 30 mg/day was also started at admission; however, it was suspended a few days before skin biopsies (three days in the first procedure and five days in the second procedure). After the exclusion of other causes, prednisolone 40 mg/day was restarted, with marked improvement of skin lesions and hypereosinophilia observed (Table 1). Ulcerative lesions also evolved favorably; however, two lesions required a surgical approach by Plastic Surgery for closure. A presumptive diagnosis of idiopathic HES was made. The patient was discharged with complete resolution of skin lesions and under a tapering course of prednisone, with no further relapsing of the skin lesions.

## Discussion

This case describes a patient on chronic treatment with rituximab who presented with idiopathic HES and exuberant dermatologic manifestations. Only a few cases of idiopathic HES with cutaneous manifestations in patients on rituximab treatment have been reported [6,7]. Besides its rarity, this case illustrates the need for a systematic approach in patients who meet the criteria for hypereosinophilia to exclude other diagnoses, such as infections, neoplasms, and immune-mediated disorders.

As an immunocompromised patient with an enriched epidemiological context, an infectious cause was a concerning hypothesis. Due to the cutaneous manifestations, infection with *Burkholderia pseudomallei *or parasites (such as Strongyloides or Toxocara) was investigated [8,9]. An extensive microbiological and serological workup was performed, and no evidence of infection was registered. No significant response to broad-spectrum antimicrobial treatment and empiric ivermectin administration was observed, so infectious etiology was not probable. 

A thorough investigation of occult neoplasm was also conducted. The whole-body CT scan showed no evidence of a solid neoplasm, and there were no significant findings from recent endoscopic studies conducted before hospitalization. Hematologic malignancy was discarded after bone marrow studies and peripheral blood immunophenotyping, particularly for eosinophilic leukemia. Both skin biopsies also did not show any signs of malignancy, such as skin T-cell lymphoma. 

Immune-mediated disorders associated with hypereosinophilia were considered. There was no evidence of eosinophilic vasculitis or immune-mediated skin disorders, such as psoriasis and pityriasis rubra, according to histological findings in skin biopsies [2]. It should be noted that although rituximab is a therapeutic option for some forms of eosinophilic vasculitis, it is more effective in treating pathophysiological mechanisms mediated by B-cells and autoantibodies. Therefore, we can hypothesize that the absence of eosinophilic vasculitis could hardly be masked by previous rituximab treatment alone [10,11]. Hypersensitivity reactions to rituximab with extensive skin involvement have been reported, such as Stevens-Johnson syndrome and drug reaction with eosinophilia and systemic symptoms (DRESS) [6,7]. However, neither the subacute evolution nor the documented cutaneous lesions were suggestive of these disorders. 

After excluding these mentioned diagnoses, idiopathic HES presented as the most probable diagnosis, attending to hypereosinophilia and skin damage not explained by another mechanism. Although slight eosinophilic inflammation was documented in skin biopsies, no significant organ infiltration or damage by eosinophils was observed. This disparity between hypereosinophilia and histological findings may be due to previous corticosteroid treatment before the biopsies were performed, which could have limited diagnostic sensitivity. The thorough exclusion of alternative diagnoses and the excellent response to systemic corticosteroid treatment support the diagnosis. 

Once blood eosinophilia is suppressed and symptoms are controlled, daily glucocorticoid doses are reduced to the lowest dose that maintains control of the eosinophil count. However, a second steroid-sparing agent should be considered, especially in this patient with concomitant rheumatoid arthritis, such as mepolizumab, an anti-IL-5 agent, along with a disease-modifying agent to control symptoms related to the previously known articular inflammatory disease [12]. 

## Conclusions

This article presents a rare case of idiopathic HES in a patient treated with rituximab. A systematic approach is needed for patients with hypereosinophilia to ensure an accurate diagnosis, which may be challenging in immunosuppressed patients.
